# Listening to real-world sounds: fMRI data for analyzing connectivity networks

**DOI:** 10.1016/j.dib.2019.104411

**Published:** 2019-08-17

**Authors:** Po-Chih Kuo, Yi-Li Tseng, Karl Zilles, Summit Suen, Simon B. Eickhoff, Juin-Der Lee, Philip E. Cheng, Michelle Liou

**Affiliations:** a Institute of Statistical Science, Academia Sinica, Taipei, Taiwan; b Department of Electrical Engineering, Fu Jen Catholic University, New Taipei City, Taiwan; c Institute of Neuroscience and Medicine (INM-1), Research Centre Jülich, Jülich, Germany; d Institute of System Neuroscience, Medical Faculty, Heinrich Heine University Düsseldorf, Düsseldorf, Germany; e Institute of Neuroscience and Medicine (INM-7), Research Centre Jülich, Jülich, Germany; f Graduate Institute of Business Administration, National Chengchi University, Taipei, Taiwan

**Keywords:** fMRI, Connectivity networks, Real-world, Auditory

## Abstract

There is a growing interest in functional magnetic resonance imaging (fMRI) studies on connectivity networks in the brain when subjects are under exposure to natural sensory stimulation. Because of a complicated coupling between spontaneous and evoked brain activity under real-world stimulation, there is no critical mapping between the experimental inputs and corresponding brain responses. The dataset contains auditory fMRI scans and T1-weighted anatomical scans acquired under eyes-closed and eyes-open conditions. Within each scanning condition, the subject was presented 12 different sound clips, including human voices followed by animal vocalizations. The dataset is meant to be used to assess brain dynamics and connectivity networks under natural sound stimulation; it also allows for empirical investigation of changes in fMRI responses between eyes-closed and eyes-open conditions, between animal vocalizations and human voices, as well as between the 12 different sound clips during auditory stimulation. The dataset is a supplement to the research findings in the paper “Brain dynamics and connectivity networks under natural auditory stimulation” published in NeuroImage.

**Table:** Specifications Table

Subject	Neuroscience
Specific subject area	Neuroimaging
Type of data	Image
How data were acquired	Data were acquired using a 3T MAGNETOM Skyra scanner (Siemens Healthcare, Erlangen, Germany) and a standard 20-channel head-neck coil.
Data format	Raw
Parameters for data collection	EPI images: TR = 2000 ms; TE = 30 ms; flip angle = 84°; 35 slices with slice thickness = 3.4 mm; FOV = 192 mm; voxel size = 3 × 3 × 3.74mm. T1 images: TR = 2530 ms; TE = 3.30 ms; flip angle = 7°; 192 slices; FOV = 256 mm; voxel size = 1 × 1 × 1mm.
Description of data collection	Data were collected from 40 subjects. The subjects were instructed to listen passively to the sound stimuli under the eyes-closed condition and then under the eyes-open condition. Each condition comprised human voices followed by animal vocalizations.
Data source location	Institution: Institute of Statistical Science, Academia Sinica City/Town/Region: Taipei City Country: Taiwan Latitude and longitude (and GPS coordinates) for collected samples/data: 24°59′12.8″N 121°34′34.5″E
Data accessibility	Repository name: Mendeley Data identification number: 10.17632/9x324z2x3b Direct URL to data: https://data.mendeley.com/datasets/9x324z2x3b
Related research article	Author's name: Po-Chih Kuo, Yi-Li Tseng, Karl Zilles, Summit Suen, Simon B. Eickhoff, Juin-Der Lee, Philip E. Cheng, Michelle Liou Title: Brain Dynamics and Connectivity Networks Under Natural Auditory Stimulation Journal: NeuroImage DOI: https://doi.org/10.1016/j.neuroimage.2019.116042

**Table:** 

**Value of the data** • The dataset can be used for assessing reproducible fMRI time courses under real-life situations. • The dataset can be used for investigating connectivity networks under natural sound stimulation. • The dataset can be used for decoding mental states during hearing animal vocalizations and human voices.

## 1 Data

The fMRI scans were acquired when subjects were under exposure to natural auditory stimulation. The same auditory stimulation was repeated twice under eyes-closed followed by eyes-open conditions. The released dataset contains the following items: (1) T1-weighted MPRAGE anatomical images with skull stripped, (2) 4D MRI images in analyzed format (i.e., fMRI data), (3) the onset time and duration of each sound clip during the experiment, and (4) sound stimuli used in the experiment.

## 2 Experimental design, materials, and methods

### 2.1 Participants and experimental procedure

**Fig. 1:**
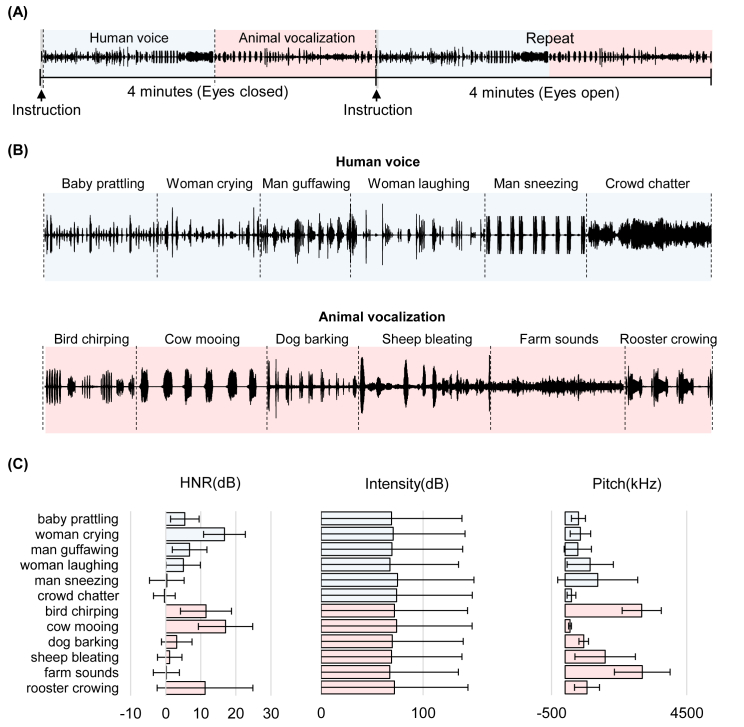
Stimuli and experimental paradigm: (A) The 8-min task began with an eye-closed condition (4 min duration) followed by an eye-open condition (4 min duration). The sounds were played to the subjects under each condition comprised of human voices and animal vocalizations. (B) The sound clips comprised six types of human voices, including a baby prattling, a woman crying, a man guffawing, a woman laughing, a man sneezing, and a crowd chattering. This was followed by six types of animal vocalizations, including a bird chirping, a cow mooing, a dog barking, a sheep bleating, various farm sounds, and a rooster crowing. The duration of each sound and its order of appearance were randomly determined for each subject. (C) No significant differences were observed between human and animal sounds in terms of acoustic features; for example, harmonics-to-noise ratio (HNR), intensity, and pitch.

Forty healthy adults (19 males, average age: 22.76 ± 3.25 years) were recruited for the experiment. The subjects were instructed to listen passively to the sound stimuli, which were presented using high-quality MR-compatible insert earphones under the eyes-closed condition (4 min duration) and then under the eyes-open condition (4 min duration). The duration of the entire experiment was 8 min, as shown in Fig. 1(A). Under the eye-open condition, the subjects were instructed to keep their eyes fixed on a central cross throughout the scanning condition. Each condition (eyes-closed or eyes-open) comprised 6 clips of human voices followed by 6 clips of animal vocalizations.

### 2.2 Stimuli

As shown in Fig. 1(B), the sound clips comprised human voices (baby prattling, woman crying, man guffawing, woman laughing, man sneezing, and crowd chatter) and animal vocalizations (bird chirping, cow mooing, dog barking, sheep bleating, rooster crowing, and actual sounds from a farm). The sound clips were collected from a collaborative database (www.freesound.org) and the PacDV website (www.pacdv.com). Each type of sounds was normalized to a similar intensity (70.81 ± 2.591dB) and presented using the Psychophysics Toolbox (www.psychtoolbox.org). The length of each clip and its order of appearance in the experiment were randomly determined within the [16, 26]-secs interval for each subject. Human voices were always presented before the animal vocalizations, as shown in Fig. 1(A). The 4-min sound track was first presented with the eyes-closed, and then repeated once with the eyes-open.

### 2.3 Acoustic features of stimuli

Fig. 1(C) presents the three acoustic features (harmonics-to-noise ratio (HNR), intensity, and pitch), which were calculated from the sound stimuli using Praat software (www.praat.org). MIRtoolbox was also used to compute other features, including zero crossing rate, energy, average frequency of events, tempo, energy above cut-off frequency, energy below cut-off frequency, centroid, spread, skewness, kurtosis, flatness of spectral distribution, spectrum entropy, and silence ratio [1]. HNR was used to measure the ratio of the strength of periodic and noisy components. It was calculated as follows:

$$\text{HNR}=10\;\log_{10}\frac{k}{\left(1-k\right)}$$

where *k* is the strength of the strongest periodic component. Intensity was calculated using the mean amplitude of the signals, whereas the pitch was calculated using an autocorrelation method. The other features reflect the rhythm and timbre of the sounds. Energy was estimated from the root-mean-square of the amplitude. Tempo was calculated by detecting periodicities in the event detection curve. Spectral distribution was described using statistical measures, including centroid, spread, skewness, kurtosis, flatness, and entropy. The silence ratio was used to measure the percentage of frames with energy levels below the mean energy across all frames. Note that there was no significant difference between the human voices and animal vocalizations in terms of these 16 acoustic features (with a *p*-value ranging from 0.12 to 0.86).

### 2.4 Acquisition procedure

MR scans were acquired using a 3T MAGNETOM Skyra scanner (Siemens Healthcare, Erlangen, Germany) and a standard 20-channel head-neck coil. A total of 35 slices were acquired every 2 s using a single-shot gradient EPI, which provided whole-head coverage (TR = 2000 ms; TE = 30 ms; flip angle = 84°; 35 slices with slice thickness = 3.4 mm; FOV = 192 mm; voxel size = 3 × 3 × 3.74 mm). A further 192 slices of T1-weighted anatomical images were also collected for image co-registration (TR = 2530 ms; TE = 3.30 ms; flip angle = 7°; 192 slices; FOV = 256 mm; voxel size = 1 × 1 × 1 mm).

### 2.5 Data preprocessing

The data can be preprocessed using SPM12 (http://www.fil.ion.ucl.ac.uk/spm). Low-resolution EPI images from each subject can be realigned in six dimensions using rigid body transformation (translation did not exceed 2 mm and rotation corrections did not exceed 2°). The T1-weighted high-resolution image volume can be co-registered to the mean of the realigned EPI image and spatially normalized using a voxel size of 2 × 2 × 3 mm to the standard space, as defined by Montreal Neurological Institute (MNI) T1-weighted template. The signal-to-noise ratio of the functional images can be enhanced via spatial smoothing using a 4-mm full-width-at-half-maximum Gaussian filter. The trends of temporal blood oxygen level-dependent (BOLD) signals caused by drifts in the magnetic field can be corrected by removing the global mean of the signals [2], [3]. In intra-subject analysis, the fMRI time series can be divided into two replicates: the eyes-closed condition and eyes-open condition. For more details about the data and analysis methods, please see the original publication in Ref. [4].
